# HER2-HER3 Heterodimer Quantification by FRET-FLIM and Patient Subclass Analysis of the COIN Colorectal Trial

**DOI:** 10.1093/jnci/djz231

**Published:** 2019-12-18

**Authors:** Paul R Barber, Gregory Weitsman, Katherine Lawler, James E Barrett, Mark Rowley, Manuel Rodriguez-Justo, David Fisher, Fangfei Gao, Iain D C Tullis, Jinhai Deng, Louise Brown, Richard Kaplan, Daniel Hochhauser, Richard Adams, Timothy S. Maughan, Borivoj Vojnovic, Anthony C C Coolen, Tony Ng

**Affiliations:** 1 UCL Cancer Institute, Paul O’Gorman Building, University College London, London, UK; 2 Richard Dimbleby Laboratory of Cancer Research, School of Cancer & Pharmaceutical Sciences, King’s College London, London, UK; 3 Institute for Mathematical and Molecular Biomedicine, King’s College London, Guy’s Medical School Campus, London, UK; 4 Saddle Point Science Ltd, London, UK; 5 MRC Clinical Trials Unit at UCL, Institute of Clinical Trials & Methodology, London, UK; 6 Department of Oncology, Cancer Research UK and Medical Research Council Oxford Institute for Radiation Oncology, University of Oxford, Oxford, UK; 7 School of Medicine, Cardiff University, Cardiff, UK; 8 Breast Cancer Now Research Unit, Department of Research Oncology, Guy’s Hospital King’s College London, London, UK

## Abstract

**Background:**

The phase III MRC COIN trial showed no statistically significant benefit from adding the EGFR-target cetuximab to oxaliplatin-based chemotherapy in first-line treatment of advanced colorectal cancer. This study exploits additional information on HER2-HER3 dimerization to achieve patient stratification and reveal previously hidden subgroups of patients who had differing disease progression and treatment response.

**Methods:**

HER2-HER3 dimerization was quantified by fluorescence lifetime imaging microscopy in primary tumor samples from 550 COIN trial patients receiving oxaliplatin and fluoropyrimidine chemotherapy with or without cetuximab. Bayesian latent class analysis and covariate reduction was performed to analyze the effects of HER2-HER3 dimer, *RAS* mutation, and cetuximab on progression-free survival and overall survival (OS). All statistical tests were two-sided.

**Results:**

Latent class analysis on a cohort of 398 patients revealed two patient subclasses with differing prognoses (median OS = 1624 days [95% confidence interval [CI] = 1466 to 1816 days] vs 461 days [95% CI = 431 to 504 days]): Class 1 (15.6%) showed a benefit from cetuximab in OS (hazard ratio = 0.43, 95% CI = 0.25 to 0.76, *P* = .004). Class 2 showed an association of increased HER2-HER3 with better OS (hazard ratio = 0.64, 95% CI = 0.44 to 0.94, *P* = .02). A class prediction signature was formed and tested on an independent validation cohort (n = 152) validating the prognostic utility of the dimer assay. Similar subclasses were also discovered in full trial dataset (n = 1630) based on 10 baseline clinicopathological and genetic covariates.

**Conclusions:**

Our work suggests that the combined use of HER dimer imaging and conventional mutation analyses will be able to identify a small subclass of patients (>10%) who will have better prognosis following chemotherapy. A larger prospective cohort will be required to confirm its utility in predicting the outcome of anti-EGFR treatment.

The selection of patients who are likely to benefit from treatment with an EGFR inhibitor with first-line chemotherapy for metastatic colorectal cancer (mCRC) remains controversial. Two major trials have compared the addition of cetuximab or bevacizumab for patients with *KRAS* wild-type (WT) colorectal cancer (1–3). FIRE-3 showed a statistically significant survival benefit from cetuximab in contrast to the US-based CALGB 0405 study, leading to intense debate (4). Results show markedly improved overall survival (OS) compared with older trials, partially driven by the selection of the better prognosis *KRAS* WT population. The presence of any extended RAS mutation (5) was demonstrated to exclude patients from benefit of panitumumab in the PRIME trial; this is now enshrined in license (6).

Other molecular factors also influence responsiveness to the addition of an EGFR inhibitor: the presence of a *BRAF* mutation and low expression of key EGFR ligands, epiregulin (*EREG*) or amphiregulin, both predict a lack of benefit (7,8). The primary tumor’s site of origin is also important. Tumors arising from the right, midgut derived, colon, falling in the arterial supply of the superior mesenteric artery are more frequently methylated (with resulting low expression of EGFR ligands) (9–11), more often have mismatch repair deficiency, and carry a *RAF* mutation (12–14). Left-sided cancers more often exhibit those features of responsiveness to EGFR treatment, namely high ligand expression and *RAS* and *RAF* WT. Initial reports also indicated that *PIK3CA* mutations may be associated with diminished responsiveness, but these conclusions were from small studies (2–11 patients with *PIK3CA* mutations) (15,16). Larger studies did not show a statistically significant difference (17,18), except for one study showing that *PIK3CA* exon 20 mutation confers a poorer outcome (19). Despite all this, reliable methods for the identification of patients who may benefit from EGFR antibody therapy remain elusive.

In this article, we describe a novel approach to this problem. It is known that HER (ErbB)-mediated signaling is initiated following dimerization between the same (homodimerization) or different HER family members (EGFR, ErbB/HER1-4) (20). Dimers containing HER3, especially the HER2-HER3 heterodimer, have been shown to provide the most potent proliferative signal to cancer cells (21). Recently, we showed in preclinical experiments the HER2-HER3 can be modulated on cetuximab treatment of colorectal cancer cells (22). The same heterodimer has been demonstrated using archived primary breast cancer samples and contains statistically significant prognostic information, which is independent of that of HER2 receptor expression status (23). It is usually difficult to determine whether the receptors are forming dimers, but the technique of Förster resonance energy transfer (FRET) reports on the immediate proximity, only achieved during dimerization. The combination of FRET with time-domain fluorescence lifetime imaging microscopy (FLIM) allows the minute fluorescence signals to be detected (24). Hence, FRET-FLIM represents the most exquisitely sensitive method for determining what proportion of a receptor is dimerized.

Here we report the use of FLIM histology, a technique using FRET-FLIM as a measure of the proportion of receptors in the HER2-HER3 dimer state, a concentration-independent parameter, based on a well-established gold standard technique to probe endogenous protein-protein interactions in cells (23,25–30). In 550 patients from the MRC COIN trial (31), combining the use of HER dimer measurement and recently reported Bayesian statistical methods (32–34), we aimed to identify subclasses of patients with different prognostic outcomes.

## Materials and Methods

### Patients and Treatment

In the MRC COIN trial (ISRCTN79877428) (31), patients with histologically confirmed adenocarcinoma of the colon or rectum, including inoperable metastatic or locoregional measurable disease (RECIST v1.0), and who were fit for first-line combination chemotherapy were randomly assigned in a 1:1:1 ratio to receive the control arm of continuous oxaliplatin-based chemotherapy (A), continuous chemotherapy plus cetuximab (B), or intermittent chemotherapy (C). This study was restricted to arms A and B. Two chemotherapy regimens, XELOX or OxMdG (oxaliplatin with modified deGramont, a FOLFOX variant), were used.

### Objectives and Outcome Measures

The primary objective of the COIN A vs B comparison was to determine whether the addition of cetuximab to continuous chemotherapy resulted in improved outcome in patients with *KRAS* WT tumors. OS was calculated as time from randomization to death from any cause. Survivors were censored at the last known alive date. Progression-free survival (PFS) was calculated as the interval from randomization to first evidence of progression or death from any cause. Survivors without progression were censored at the last known alive date.

### Patient Samples and Imaging

This study was approved by the Trial Steering Committee, and FRET-FLIM was limited to those patients who had given written informed consent for “other bowel cancer research” in whom enough residual pathological specimen was available.

Patient tissue microarrays (TMAs) were retrieved from the Wales Cancer Bank and processed at King’s College London. Two consecutive slices of all TMAs underwent antigen retrieval in a Ventana BenchMark system and were stained with anti-HER3-IgG-Alexa546 (“donor” or “D” slice) and in addition with anti-HER2-IgG-Cy5 (“donor with acceptor” or “DA” slice) and mounted as described previously (26).

TMA slices were imaged on an “open” automated FLIM microscope (35). FLIM analysis was performed with the TRI2 software (v2.7.8.9, CRUK/MRC Oxford Institute for Radiation Oncology, Oxford, UK) (36–38). Autofluorescence effects were minimized with a lifetime filtering algorithm (39). The FRET efficiency for each tissue region was calculated according to FRET efficiency = 1 – (τ_DA_/τ_D_), where τ_D_ and τ_DA_ are the average lifetime of Alexa546 in the matching D and DA images, respectively. FRET efficiency (denoted: FRET) and FRET efficiency multiplied by HER3 fluorescence intensity, representing the amount of dimerized HER3 (FRET × HER3), were calculated as continuous variables (Supplementary Figure 1 available online).

The use of formol saline fixation, as opposed to neutral buffered formalin, resulted in excessive amounts of contaminating autofluorescence. These samples (292 patients) were excluded.

TMAs from the 398-patient training set and the 152-patient validation set were received and processed independently in two batches. All analysis of the training set was performed before the validation TMAs were received and was therefore performed completely blind and without knowledge of the validation set.

### Statistical Analysis

Bayesian latent class analysis (LCA) was performed using the model described by Rowley et al. (32) (ALPACA v0.2.15), which seeks to detect and map association and base hazard rate heterogeneity. This results in objective cohort stratification, driven strictly by observed and statistically significant regularities in the data. Specification of the number of latent classes and the complexities of class-dependent base hazard rates is based on Bayesian model selection. Patients were retrospectively assigned to latent groups according to maximum a posteriori class membership probability.

Covariate reduction and the generation of predictive signatures was performed by Bayesian multivariable survival analysis with repeated cross-validation and backwards elimination with the aim of reducing overfitting (33).

Kaplan-Meier plots and log-rank statistics were produced using the R “survival” package (v2.42–3, R v3.5.1). When *P* was less than .05, the result was considered statistically significant and all tests were two-sided.

## Results

**Figure 1.**
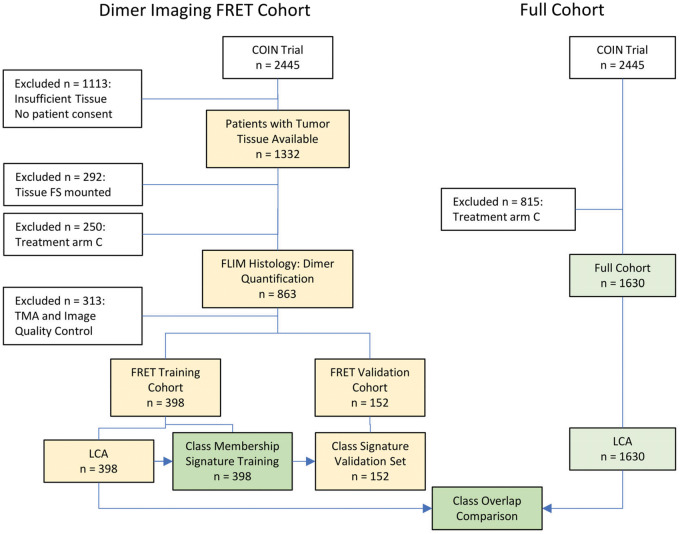
CONSORT diagram of patient selection and analysis flow for the Förster resonance energy transfer (FRET) cohorts that were subject to dimer imaging and the full cohort. Patients excluded at the Tissue microarray (TMA) and image quality control stage had insufficient tissue remaining on the slide, the tissue was of bad quality (eg, folded), or the donor and donor-acceptor areas could not be matched. Class membership revealed by Latent class analysis (LCA) on the FRET cohort of 398 was used to train a class membership signature, which was tested in the validation set of 152 patients. The concordance between the LCA on the FRET and full cohorts was assessed in a class overlap comparison. A = oxaliplatin and fluoropyrimidine chemotherapy; B = A + cetuximab; C = intermittent chemotherapy; FLIM = fluorescence lifetime imaging microscopy; FS = formol saline.

**Figure 2.**
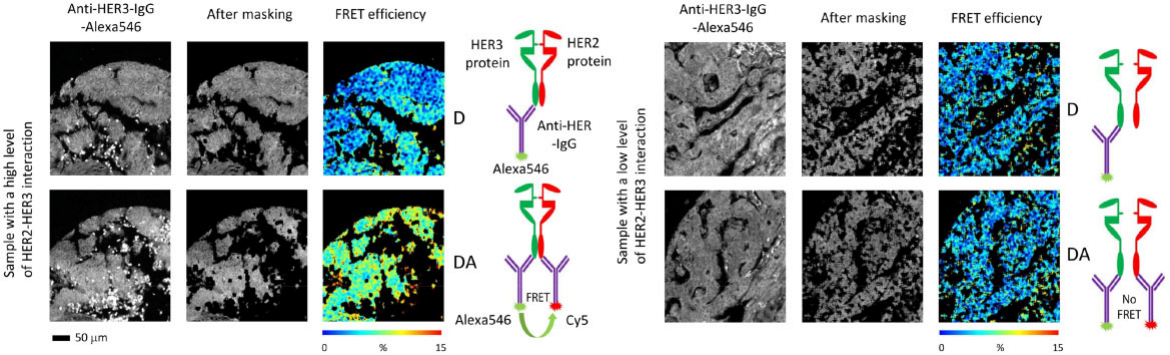
Detection of HER2-HER3 dimerization by fluorescence lifetime imaging microscopy. Förster resonance energy transfer (FRET) efficiency maps indicate degree of HER2-HER3 interaction. **Scale bar** = 50 μm. D = FRET donor only sample; DA = FRET donor + acceptor sample; IgG = Immunoglobulin G.

Tissues from two cohorts of 398 and 152 patients (the FRET training and validation cohorts, respectively) were analyzed for HER2-HER3 dimerization. All patients also formed a “full” cohort of 1630 patients. Figure 1 summarizes the patient selection for imaging and analysis, and Table 1 contains the cohort patient characteristics. A continuous distribution of FRET efficiency with a mean value of 1.6% (lower quartile, 0.18%; upper quartile, 2.7%) was recorded. Figure 2 shows typical images and FRET efficiency maps.

**Table 1.** Patient characteristics of the full and FRET cohorts*

Covariate	Full cohort	FRET training	FRET validation
No.	1630	398	152
Treatment arm = B, no. (%)	815 (50.0)	209 (52.5)	80 (52.6)
CHEMO = XELOX, no. (%)	1070 (65.6)	223 (56.0)	111 (73.0)
Age at randomization, mean (SD)	62.34 (9.79)	63.04 (9.60)	62.77 (9.12)
Sex = male, no. (%)	1069 (65.6)	278 (69.8)	95 (62.5)
Height, mean (SD), cm	170.10 (9.33)	170.14 (9.13)	169.65 (8.92)
Weight, mean (SD), kg	76.05 (15.98)	75.66 (15.63)	78.02 (17.39)
WHO performance status, mean (SD)	0.62 (0.62)	0.58 (0.59)	0.62 (0.65)
Sidedness of primary tumor, no. (%)			
Left-sided	1138 (69.8)	274 (68.8)	103 (67.8)
Right-sided	460 (28.2)	117 (29.4)	48 (31.6)
Unknown	32 (2.0)	7 (1.8)	1 (0.7)
TSTAT, no. (%)			
Local recurrence	88 (5.4)	24 (6.0)	18 (11.8)
Resected	865 (53.1)	315 (79.1)	115 (75.7)
Unresected or unresectable	677 (41.5)	59 (14.8)	19 (12.5)
Metastatic sites = polymetastatic (>3), no. (%)	71 (4.4)	17 (4.3)	6 (3.9)
Mlivonly = yes, no. (%)	368 (22.6)	98 (24.6)	34 (22.4)
Metscat, no. (%)			
Metachronous	489 (30.0)	159 (39.9)	68 (44.7)
Synchronous	1123 (68.9)	237 (59.5)	84 (55.3)
Unknown	18 (1.1)	2 (0.5)	0 (0.0)
MNODE = yes, no. (%)	720 (44.2)	172 (43.2)	75 (49.3)
Tumor marker: CEA value, mean (SD)	686.99 (2849.05)	374.37 (1310.56)	484.72 (1463.96)
Tumor marker: CA 19-9 value, mean (SD)	2946.00 (13052.42)	546.26 (705.36)	254.50 (152.78)
EREG Cq value, negated, mean (SD)	−3.16 (2.26)	−3.28 (2.27)	−3.10 (2.01)
AREG Cq value, negated, mean (SD)	−2.82 (1.60)	−2.84 (1.58)	−2.73 (1.65)
*KRAS*, no. (%)			
Mutation	570 (35.0)	165 (41.5)	73 (48.0)
Wild type	744 (45.6)	225 (56.5)	78 (51.3)
Unknown	316 (19.4)	8 (2.0)	1 (0.7)
*NRAS*, no. (%)			
Mutation	51 (3.1)	17 (4.3)	5 (3.3)
Wild type	1259 (77.2)	374 (94.0)	147 (96.7)
Unknown	320 (19.6)	7 (1.8)	0 (0.0)
MSI, no. (%)			
MSI	45 (2.8)	15 (3.8)	5 (3.3)
Stable	977 (59.9)	314 (78.9)	132 (86.8)
Unknown	608 (37.3)	69 (17.3)	15 (9.9)
*PIK3CA*, no. (%)			
Mutation	156 (9.6)	49 (12.3)	26 (17.1)
Wild type	1107 (67.9)	334 (83.9)	126 (82.9)
Unknown	367 (22.5)	15 (3.8)	0 (0.0)
*BRAF*, no. (%)			
Mutation	102 (6.3)	29 (7.3)	11 (7.2)
Wild type	1192 (73.1)	360 (90.5)	141 (92.8)
Unknown	336 (20.6)	9 (2.3)	0 (0.0)
ADJCH, no. (%)			
>1 mo and <6 mo ago	68 (4.2)	24 (6.0)	8 (5.3)
>6 mo ago	261 (16.0)	82 (20.6)	33 (21.7)
No	1218 (74.7)	269 (67.6)	99 (65.1)
Yes, unspecified	83 (5.1)	23 (5.8)	12 (7.9)
Sum of longest diameter, mean (SD)	106.65 (85.19)	103.88 (81.57)	96.05 (70.83)
Platelet count, mean (SD)	356.31 (132.62)	346.38 (119.65)	329.67 (132.96)
Neutrophil count, mean (SD)	6.29 (3.58)	5.77 (2.64)	5.96 (4.94)
White blood cell count, mean (SD)	8.98 (3.99)	8.51 (3.06)	8.26 (2.92)
Alkaline phosphatase, mean (SD)	191.67 (176.79)	180.77 (171.20)	170.72 (145.04)
Pain at baseline (CTC grade), mean (SD)	0.55 (0.74)	0.49 (0.72)	0.38 (0.66)
Anorexia at baseline, CTC grade, mean (SD)	0.23 (0.54)	0.20 (0.50)	0.12 (0.37)
Vomiting at baseline, CTC grade, (SD)	0.04 (0.24)	0.03 (0.21)	0.02 (0.14)
Lethargy at baseline, CTC grade, mean (SD)	0.49 (0.65)	0.44 (0.60)	0.38 (0.61)
Hemoglobin at baseline, CTC grade, mean (SD)	0.25 (0.56)	0.20 (0.47)	0.12 (0.40)
Nail changes at baseline, CTC grade, mean (SD)	0.01 (0.09)	0.02 (0.14)	0.00 (0.00)

* ADJCH = Adjuvant chemotherapy; AREG = amphiregulin; CA = cancer antigen 19-9; CEA = Carcinoembryonic antigen; CHEMO = Chemotherapy; CTC = Common toxicity criteria; EREG = epiregulin; MNODE = Nodal metastases status; MSI = Microsatellite stability status; TSTAT = Baseline tumour status; WHO = World Health Organisation; XELOX = Oxaliplatin and capecitabine chemotherapy.

LCA was performed on the FRET training cohort for both outcomes using a minimal 4 covariates: FRET; FRET × HER3 (because HER protein concentration information is independent of dimer (23)); treatment arm (to give the algorithm the ability to detect groups with different responses); and *RAS* mutation status (because of its known association with cetuximab treatment).

**Figure 3.**
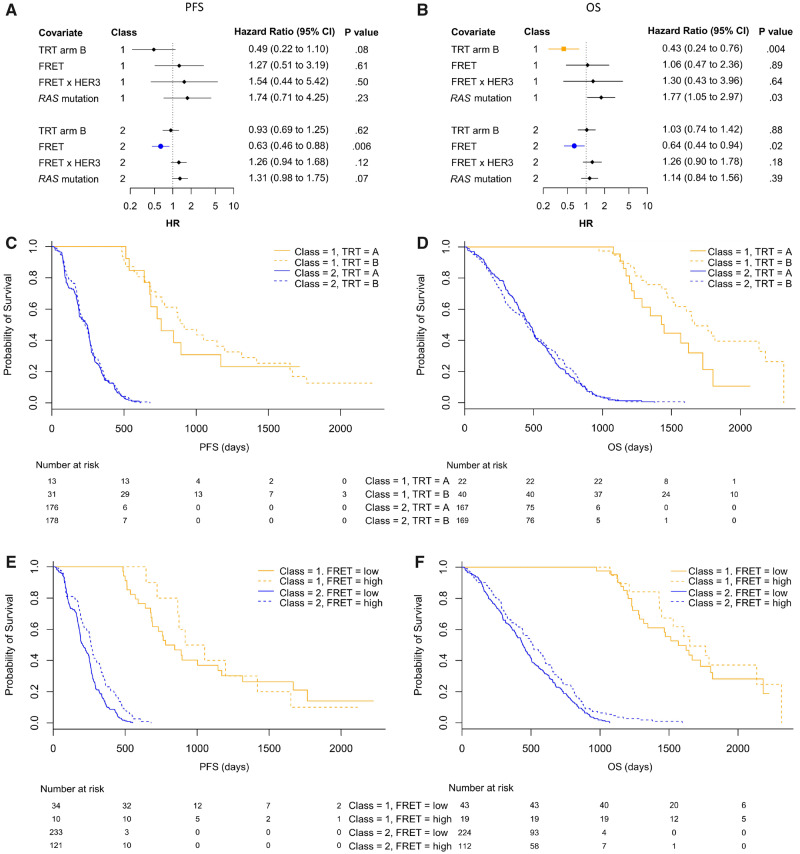
Multivariable latent class analysis of the Förster resonance energy transfer (FRET) cohort. **A and B**) Tables of covariate-associated hazard ratios (HR, diamonds, squares and circles) for the two discovered classes. For those in Class 1, treatment arm (TRT) B (cetuximab) was protective (for overall survival [OS], **squares**). For those in Class 2, a high FRET HER2-HER3 dimer score was protective (**circles**). CI = confidence interval. **C and D**) Survival curves split by class and TRT to show potential prognostic and predictive value for OS and progression-free survival (PFS). Log-rank *P* values for prognostic and predictive splits show that FRET-based LCA with 398 patients has a clear prognostic (log-rank *P* < .001) and potential predictive value: cetuximab (TRT B) was effective for patients in OS Class 1 (log-rank *P* = .05). **E and F**) Survival curves split by class and FRET efficiency. The statistically significant hazard ratio associated with FRET in Class 2 is demonstrated. Patients in Class 2 have a better outcome if their HER2-HER3 FRET efficiency is in the upper tertile (PFS log-rank *P* < .001, OS log-rank *P* = .02). All statistical tests were two-sided.

We report evidence of two novel latent classes in the 398-patient training set with both PFS and OS analysis. The hazard ratios (HR) assigned to each covariate for each class is shown in Figure 3, A and B. Based on PFS, 44 of 398 (11.1%) patients were retrospectively assigned to Class 1, the remainder to Class 2; for OS, 62 of 398 (15.6%) patients were assigned to Class 1. Figure 3, C and D shows Kaplan-Meier plots split by class and treatment (TRT). Class 1 patients had a better prognosis (median OS = 1624 days, 95% CI = 1466 to 1816 days vs 461 days, 95% CI = 431 to 504 days) and a predictive response to cetuximab that was more pronounced in OS: Class 1 TRT HR = 0.43, 95% CI = 0.25 to 0.76, log-rank *P*  = .003 (median OS = 1447 days vs 1668 days; difference = 221 days; see Supplementary Methods [available online] for more details). This is statistically significantly larger than among all patients in the cohort (median OS = 505 days vs 581 days; difference = 76 days).

The second and consistently larger group (Class 2) did not show a statistically significant benefit from cetuximab (PFS: HR = 0.93, 95% CI = 0.69 to 1.25, *P* = .62; OS: HR = 1.03, 95% CI = 0.74 to 1.42, *P* = .88); however, increased FRET efficiency was associated with improved outcome (PFS: HR = 0.63, 95% CI = 0.46 to 0.88, *P* = .006; OS: HR = 0.64, 95% CI = 0.44 to 0.94, *P* = .02) (Figure 3, A and C). Figure 3, E and F shows Kaplan-Meier curves split by class and FRET demonstrating the benefit of cetuximab to those with a high FRET score. FRET × HER3 did not have a statistically significant HR.

Table 2 shows the characteristics of the patient classes and gives an indication of which parameters may be useful in a prospective patient classifier (*P* < .05): FRET (Supplementary Figure 2 available online), liver-only metastases, *PIK3CA* mutation status, RECIST sum of longest diameter, neutrophil count, white blood cell count, pain at baseline, hemoglobin, and alkaline phosphatase.

**Table 2.** Patient characteristics of the two discovered latent classes for PFS and OS from the FRET cohort

Covariate	PFS Class 1	PFS Class 2	*P* *	OS Class 1	OS Class 2	*P* *
No.	44	354		62	336	
Treatment arm = B (%)	31 (70.5)	178 (50.3)	.02	40 (64.5)	169 (50.3)	.05
CHEMO = XELOX (%)	20 (45.5)	203 (57.3)	.18	33 (53.2)	190 (56.5)	.73
Age at randomization, mean (SD)	61.64 (10.65)	63.22 (9.47)	.30	61.65 (11.71)	63.30 (9.16)	.21
Sex = male, no. (%)	29 (65.9)	249 (70.3)	.67	41 (66.1)	237 (70.5)	.59
Height, mean (SD), cm	170.16 (8.78)	170.13 (9.19)	.99	169.87 (8.95)	170.19 (9.18)	.80
Weight, mean (SD), kg	74.40 (12.93)	75.82 (15.94)	.57	74.92 (14.11)	75.80 (15.91)	.68
WHO performance status, mean (SD)	0.43 (0.59)	0.60 (0.59)	.07	0.45 (0.56)	0.61 (0.59)	.06
Sidedness of primary tumor, no. (%)			.22			.93
Left-sided	35 (79.5)	239 (67.5)		44 (71.0)	230 (68.5)	
Right-sided	9 (20.5)	108 (30.5)		17 (27.4)	100 (29.8)	
Unknown	0 ( 0.0)	7 ( 2.0)		1 ( 1.6)	6 ( 1.8)	
Baseline tumor status, no. (%)			.006			.01
Local recurrence	7 (15.9)	17 ( 4.8)		6 ( 9.7)	18 ( 5.4)	
Resected	34 (77.3)	281 (79.4)		54 (87.1)	261 (77.7)	
Unresected or unresectable	3 ( 6.8)	56 (15.8)		2 ( 3.2)	57 (17.0)	
Metastatic sites = polymetastatic >3, no. (%)	0 ( 0.0)	17 ( 4.8)	.28	1 ( 1.6)	16 ( 4.8)	.43
Liver-only metastases = yes, no. (%)	17 (38.6)	81 (22.9)	.04	24 (38.7)	74 (22.0)	.008
Timing of metastases, no. (%)			.88			.70
Metachronous	18 (40.9)	141 (39.8)		27 (43.5)	132 (39.3)	
Synchronous	26 (59.1)	211 (59.6)		35 (56.5)	202 (60.1)	
Unknown	0 ( 0.0)	2 ( 0.6)		0 ( 0.0)	2 ( 0.6)	
Nodal metastases status = yes, no. (%)	18 (40.9)	154 (43.5)	.87	24 (38.7)	148 (44.0)	.52
Tumor marker: CEA value, mean (SD)	274.76 (641.77)	384.72 (1361.65)	.67	594.24 (2987.25)	339.65 (774.76)	.24
Tumor marker: CA 19-9 value, mean (SD)	278.00 (382.52)	586.50 (740.01)	.49	719.00	538.41 (720.93)	
EREG Cq value, negated, mean (SD)	−2.90 (2.09)	−3.32 (2.29)	.31	−2.97 (2.55)	−3.33 (2.23)	.32
AREG Cq value, negated, mean (SD)	−2.65 (1.59)	−2.86 (1.58)	.46	−2.69 (1.53)	−2.86 (1.59)	.48
*KRAS* mutation status, no. (%)			.39			.54
Mutation	14 (31.8)	151 (42.7)		22 (35.5)	143 (42.6)	
Wild type	29 (65.9)	196 (55.4)		39 (62.9)	186 (55.4)	
Unknown	1 ( 2.3)	7 ( 2.0)		1 ( 1.6)	7 ( 2.1)	
*NRAS* mutation status, no. (%)			.50			.46
Mutation	1 ( 2.3)	16 ( 4.5)		2 ( 3.2)	15 ( 4.5)	
Wild type	43 (97.7)	331 (93.5)		60 (96.8)	314 (93.5)	
Unknown	0 ( 0.0)	7 ( 2.0)		0 ( 0.0)	7 ( 2.1)	
Microsatellite stability status, no. (%)			.53			.78
MSI	2 ( 4.5)	13 ( 3.7)		2 ( 3.2)	13 ( 3.9)	
Stable	37 (84.1)	277 (78.2)		51 (82.3)	263 (78.3)	
Unknown	5 (11.4)	64 (18.1)		9 (14.5)	60 (17.9)	
*PIK3CA* mutation status, no. (%)			.37			.009
Mutation	6 (13.6)	43 (12.1)		14 (22.6)	35 (10.4)	
Wild type	38 (86.4)	296 (83.6)		48 (77.4)	286 (85.1)	
Unknown	0 ( 0.0)	15 ( 4.2)		0 ( 0.0)	15 ( 4.5)	
*BRAF* mutation status, no. (%)			.54			.89
Mutation	5 (11.4)	24 ( 6.8)		4 ( 6.5)	25 ( 7.4)	
Wild type	38 (86.4)	322 (91.0)		57 (91.9)	303 (90.2)	
Unknown	1 ( 2.3)	8 ( 2.3)		1 ( 1.6)	8 ( 2.4)	
Adjuvant chemotherapy, no. (%)			.71			.83
>1 mo and <6 mo ago	3 ( 6.8)	21 ( 5.9)		4 ( 6.5)	20 ( 6.0)	
>6 mo ago	10 (22.7)	72 (20.3)		13 (21.0)	69 (20.5)	
No	27 (61.4)	242 (68.4)		43 (69.4)	226 (67.3)	
Yes, unspecified	4 ( 9.1)	19 ( 5.4)		2 ( 3.2)	21 ( 6.2)	
Sum of longest diameter, mean (SD)	81.66 (74.30)	106.67 (82.11)	.05	68.39 (52.90)	110.49 (84.28)	<.001
Platelet count, mean (SD)	348.34 (109.90)	346.13 (120.95)	.91	341.32 (99.13)	347.31 (123.18)	.72
Neutrophil count, mean (SD)	4.76 (1.69)	5.90 (2.71)	.007	5.10 (1.73)	5.90 (2.76)	.03
White blood cell count, mean (SD)	7.50 (2.15)	8.63 (3.14)	.02	7.98 (2.28)	8.61 (3.18)	.14
Alkaline phosphatase, mean (SD)	171.11 (207.48)	181.98 (166.44)	.69	140.52 (150.16)	188.22 (174.00)	.04
Pain at baseline, CTC grade, mean (SD)	0.32 (0.60)	0.51 (0.74)	.09	0.32 (0.65)	0.52 (0.73)	.05
Anorexia at baseline, CTC grade, mean (SD)	0.09 (0.29)	0.21 (0.51)	.13	0.16 (0.45)	0.20 (0.50)	.53
Vomiting at baseline, CTC grade, (SD)	0.00 (0.00)	0.03 (0.22)	.31	0.00 (0.00)	0.04 (0.23)	.22
Lethargy at baseline, CTC grade, mean (SD)	0.34 (0.57)	0.45 (0.60)	.24	0.32 (0.57)	0.46 (0.60)	.09
Hemoglobin at baseline, CTC grade, mean (SD)	0.09 (0.29)	0.22 (0.49)	.09	0.10 (0.30)	0.22 (0.50)	.05
Nail changes at baseline, CTC grade, mean (SD)	0.00 (0.00)	0.02 (0.15)	.31	0.00 (0.00)	0.02 (0.15)	.22
FRET: HER2-HER3 FRET efficiency, mean (SD)	0.01 (0.02)	0.02 (0.02)	.006	0.01 (0.02)	0.02 (0.03)	.10
FRET × HER3 intensity, mean (SD)	0.26 (1.37)	0.66 (1.81)	.15	0.52 (1.26)	0.64 (1.85)	.63

* Chi-squared test for categorical values or ANOVA for continuous variables, all two-sided. AREG = amphiregulin; CA = cancer antigen 19-9; CEA = Carcinoembryonic antigen; CHEMO = Chemotherapy; CTC = Common Ttoxicity Ccriteria; EREG = epiregulin; FRET = Forster resonance energy transfer dimer measurement; MNODE = Nodal metastases status; MSI = Microsatellite stability status; OS = Overall survival; TSTAT = Baseline tumour status; WHO = World Health Organisation; XELOX = Oxaliplatin and capecitabine chemotherapy.

Additional LCA was performed without the FRET parameters, and we determined that there was insufficient evidence for distinct latent groups. The HER2-HER3 FRET efficiency data therefore convey additional information.

As validation of this class structure we sought further evidence in the full COIN cohort (1630 patients, including FRET cohorts) for whom clinical and genomic data were available. To maximize the utility of any findings for patient stratification, we performed analysis with all available baseline covariates (115 covariates including missingness indicators, expanded categorical data, and TRT; see Supplementary Methods available online). These were subject to Bayesian covariate reduction against OS, and we identified a signature that combined 10 covariates (World Health Organization performance status, previous adjuvant chemotherapy status, RECIST sum of longest diameter, number of metastatic sites, *EREG*, *RAS* status [*KRAS* or *NRAS*], *BRAF* status, neutrophil count, alkaline phosphatase, and pain).

**Figure 4.**
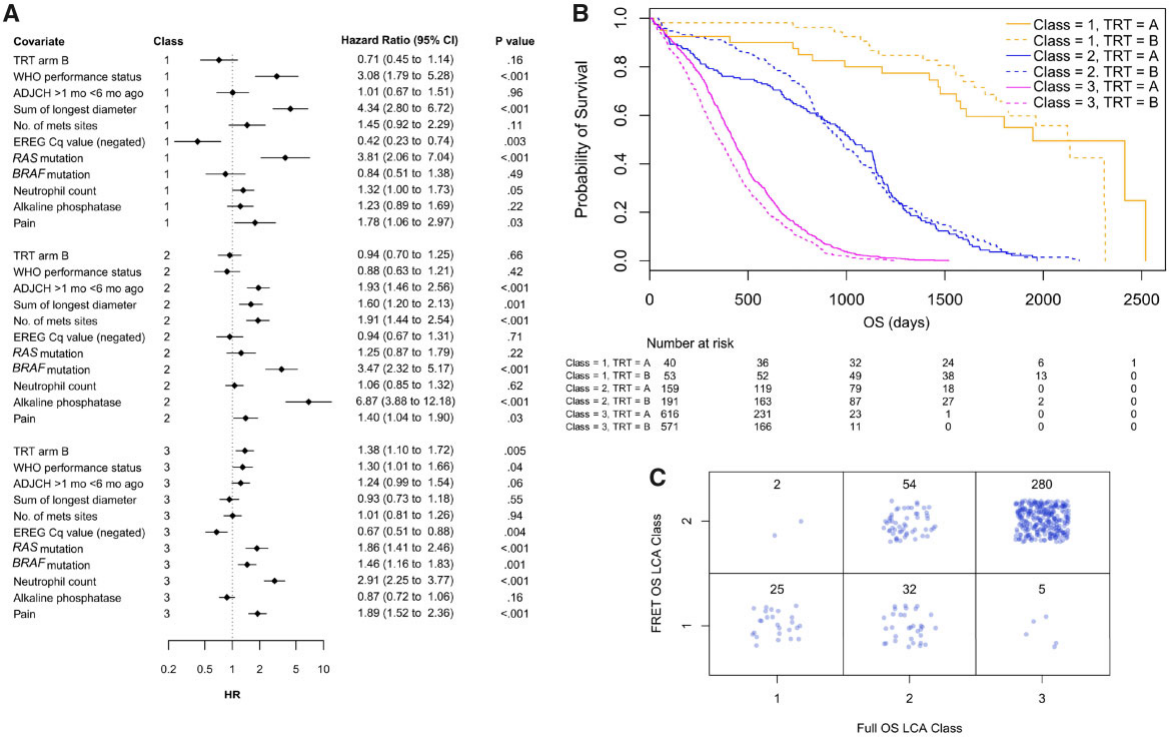
Latent class analysis (LCA) testing for similar classes within the full cohort using baseline covariates and overall survival (OS). **A**) Table of covariates and associated Hazard ratios (HR, diamonds). CI = confidence interval; TRT = Treatment. **B**) Kaplan-Meier plot split by class and treatment arm. The three classes are prognostic (log-rank *P* < .001). Class 3 predicts a treatment response (log-rank *P* < .001). **C**) LCA OS class membership comparison between the 398 Förster resonance energy transfer (FRET) cohort (two classes, Figure 3) and the overlap with the full cohort (three classes). A randomized permutations test indicates a nonrandom overlap of patients with the class sets. All statistical tests were two-sided.

These 10 covariates, plus treatment (TRT), in the full cohort were subject to OS-based LCA, which revealed that three classes were most likely (Figure 4A). Class 1 (n = 93 of 1630 patients) indicates a possibility of a positive response to cetuximab (HR = 0.71, 95% CI = 0.45 to 1.14, *P* = .16), Class 2 (n = 350 of 1630 patients) shows no response (HR = 0.94, 95% CI = 0.70 to 1.25, *P* = .66), and Class 3 (n = 1187 of 1630 patients) shows a statistically significant negative response (HR = 1.34, 95% CI = 1.10 to 1.71, *P* = .005). This is reflected in the Kaplan-Meier curve split by class and TRT (Figure 4B). PFS-based LCA did not reveal interesting classes.

To investigate the overlap in membership of individual patients between the classes of the two LCA analyses from the FRET cohort and the full cohort, the class membership table for the 398 FRET cohort patients is presented in Figure 4C. A permutations test (100 000 random permutations of 398 patients into classes in these proportions) indicated a probability of less than 1 in 100 000 for obtaining this overlap in membership by chance. LCA was also performed on the nonoverlapping set of 1232 patients (1630 minus 398), and a similar three groups were found (See Supplementary Figure 3 available online).

In the FRET cohort, there was a statistically significant association of *PIK3CA* mutation with better OS (median 875 vs 504 days, log-rank *P* = .03; Supplementary Figure 4 available online), which agrees with the observation of a higher proportion of *PIK3CA* mutant in the responding Class 1. This association was not detectable in the full cohort. A breakdown into exon 9 or exon 20 *PIK3CA* mutation groups did not reveal any statistically significant differences in PFS or OS in either cohort (FRET cohort: exon 9, n = 37 of 398; exon 20, n = 12 of 398; full cohort: exon 9, n = 106 of 1630; exon 20, n = 50 of 1630).

**Figure 5.**
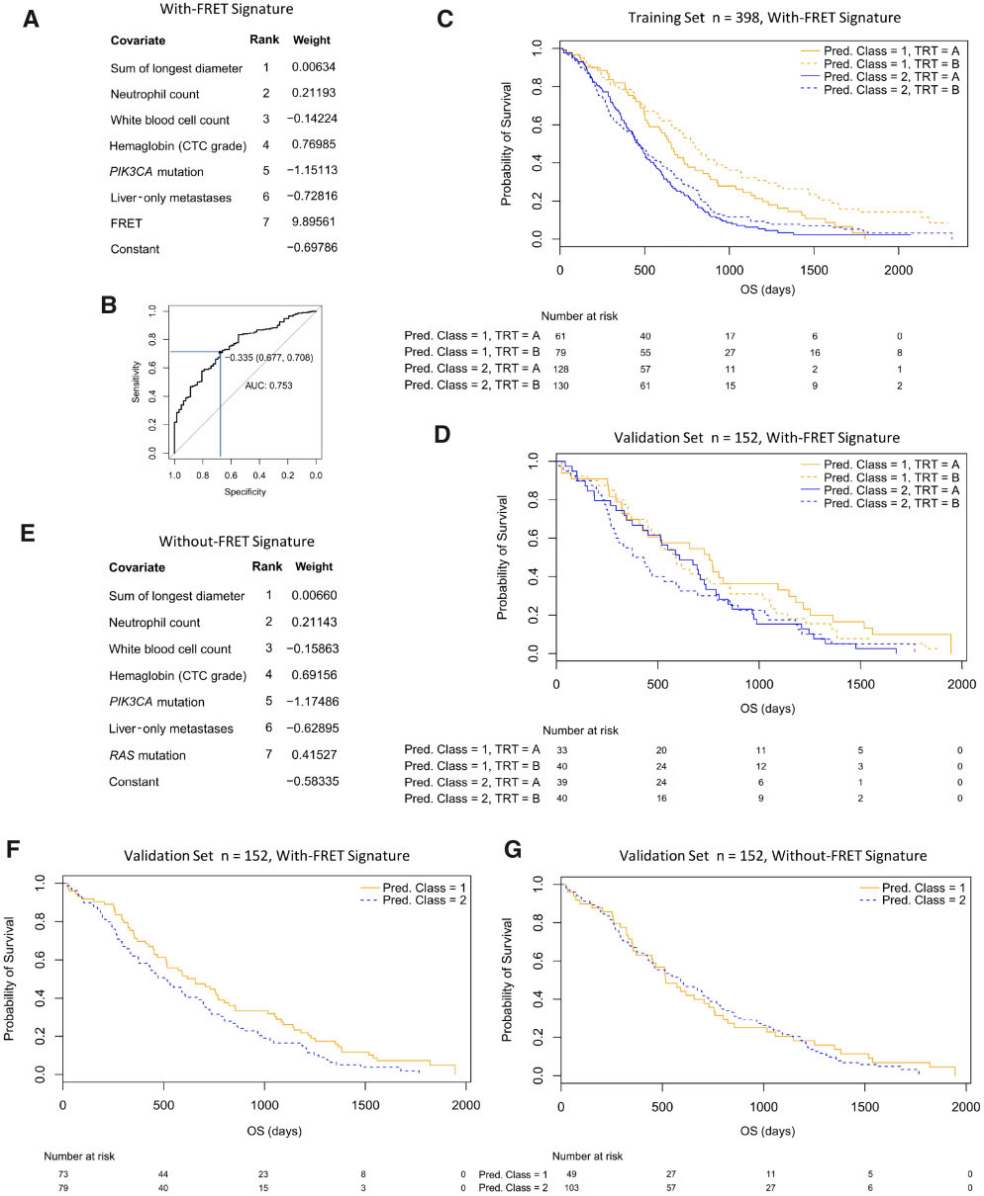
Mixed covariate class prediction signatures with and without Förster resonance energy transfer (FRET). **A**) Table of selected covariates in the with-FRET signature ranked by importance. The weight indicates how each covariate should be combined to form a class prediction score, with a constant that gives the signature a zero mean. Class 2 is associated with a signature score greater than −0.335. High FRET favors Class 2 because of its positive weight. CTC = Common toxicity criteria. **B**) Receiver operating characteristic curve for the class prediction score showing its performance in predicting the class of the 398 patients in the training set (specificity = 0.677, sensitivity = 0.708) and the optimal class threshold (−0.335). **C and D**) Survival curves split by class and treatment arm for the training set and independent validation set, respectively. **E**) Table of selected covariates in the without-FRET signature. **F and G**) Survival curves split by class for the with- and without-FRET signatures applied to the 152-validation set. FRET provides information that splits the classes (log-rank *P* = .04). Pred. = Predicted.

To form a covariate signature that may predict class membership, we performed Bayesian covariate reduction on the union of the nine covariates identified in Table 2 and the 10 prognostic baseline covariates: a total of 15 covariates. The resulting signature contained seven statistically significant covariates (RECIST sum of longest diameter, neutrophil count, white blood cell count, hemoglobin, *PIK3CA* mutation status, liver-only metastases, and FRET) with associated weights (Figure 5A).

The performance against the LCA class assignment of the 398 is shown in Figure 5B (area under curve = 0.753). The signature was used as a classifier by selecting an optimal point on the receiver operating characteristic curve (according to Youden’s index) with specificity of 0.677 and sensitivity of 0.708. The results on the 398-training set and the independent validation set of 152 are shown in Figure 5, C and D with survival curves split by class and treatment. The reclassification of the 398 patients using the new signature-based classifier clearly retains the prognostic (*P* = .001, chemo only patients) and predictive (*P* = .04) elements of the classes. In the 152-patient validation set, we again recreate the prognostic behavior (*P* = .04 [both TRT arms], *P* = .09 [chemo-only patients]).

Another signature was produced without FRET (from 14 parameters, Figure 5E), and Figure 5, F and G demonstrate that the with-FRET signature has prognostic power in the validation set, where the without-FRET signature does not. The interplay of FRET with the other covariates is explored in Supplementary Figure 5 (available online).

## Discussion

The selection of patients for EGFR-inhibitor treatment for mCRC remains difficult. With *KRAS* WT patients, the addition of EGFR-targeted treatment (cetuximab or panitumumab) to irinotecan or oxaliplatin chemotherapy (1,6,40,41) is associated with a statistically significant survival benefit in three of four phase II or III trials (1,6,41). However, the improvement of median PFS was only around 1–2 months. In the phase II OPUS trial, addition of cetuximab to FOLFOX4 resulted in a statistically significant improvement in PFS (8.3 months vs 7.2 months, *P* = .006) (41). In contrast, the NORDIC VII trial reported no benefits from adding cetuximab to oxaliplatin-based regimen (with bolus 5-FU) (40). EGFR immunohistochemistry is not a sufficient predictive factor for clinical benefit for cetuximab in the *KRAS* WT population (42,43).

Further molecular stratification by identifying novel subgroups will make a meaningful contribution towards assessing the efficacy of EGFR targeting in future clinical trials. Here we present the application of our recently improved and validated (23) FLIM histology analysis method for quantification of HER2-HER3 dimer in formalin-fixed paraffin-embedded samples from the randomized phase III MRC COIN trial. Using FLIM-based molecular imaging parameters and a recently published Bayesian statistical method (32), we have shown that there are two classes of patients with mCRC. Class 1 (10–15% of patients) had a better prognosis and benefited from addition of cetuximab to the standard chemotherapy. Within Class 2 (85–90% of patients), patients have less favorable survival (median PFS circa 7.5 months) and no benefit from cetuximab.

To validate these results, we formed a biomarker that predicts class membership by creating a novel signature of seven parameters that were predetermined by the two Bayesian latent class analyses. This was applied to the training set of 398, and we retained the predictive and prognostic elements of the smaller Class 1. Notably, the prognostic effect on survival (195 days, comparing chemotherapy only patients between Classes 1 and 2) was larger than the predictive effect (136 days, comparing Class 1 patients with or without cetuximab). Application of the signature to the completely independent validation set of 152 patients was enough to validate the prognostic (but not the predictive) utility. In addition, we found that patients exhibiting a high FRET value are more likely to be in the worst prognostic outcome subclass, Class 2 (Table 2), as reflected in the class prediction signature (Figure 5A). However, within Class 2 a high FRET value can be indicative of better outcome dependent on the other signature covariates. Importantly, the class prediction (seven-parameter) signature is entirely dependent on the inclusion of the HER2-HER3 dimer quantity.

We chose HER2-HER3 because it has been shown to be the most tumor-promoting dimer among EGFR family members due to its downstream activation of the PI3-kinase and MAPK pathways (44–46). Secondly, the mRNA expression of alternative ligands such as EREG, which has been shown to modulate the efficacy of EGFR-targeted agents in *KRAS* WT mCRCs (7), is the broadest specificity EGF-like ligand that induces the widespread phosphorylation of HER1-4 (47). Although the mechanism of this modulation is not precisely known, EREG, as opposed to EGF, can recruit HER3 into heterodimers, as reflected by its enhancement on the proliferative activity on cells coexpressing a combination of HER3 with either HER2 or HER4 (48). Thirdly, we showed by FRET-FLIM imaging an induction of HER2-HER3 dimers after cetuximab treatment in *KRAS* and *BRAF* WT colon cancer cells (22).

The additional HER2-HER3 dimer parameter as measured by FLIM may be important for the future stratification of anti-HER2 treatment combination using pertuzumab plus trastuzumab (49). Notably, HER2 activity (of prognostic signature) has been shown previously to be measurable by FLIM independently of HER2 concentration (23).

This new retrospective analysis suggests that the proportion of patients gaining benefit from cetuximab may be as small as 10% and concurs with clinical data that these patients are among those with the best baseline prognosis. HER2-HER3 FRET-FLIM provided new information enabling the statistical method to identify this latent class. These hypothesis-generating data show the potential of measurement of dimers and demonstrate the utility of FRET-FLIM to assess dimerization in formalin-fixed paraffin-embedded tissue.

Further preclinical experiments using patient-derived organoids, for example, are needed to understand the statistically significantly increased prevalence of *PIK3CA* mutations in the discovered Class 1. Previously anti-EGFR response was shown to be higher for *RAS* WT patients who expressed phosphoproteins pEGFR and pAkt (50). pAkt may in turn be linked to EGFR trafficking and degradation, and therefore treatment response, warranting further study (51). Furthermore, the predictive utility of this assay may be further enhanced by the inclusion of pre- and posttreatment dimer measurements, as we have recently demonstrated in a phase II head and neck study using an exosomal HER dimer assay (52).

In conclusion, this study demonstrates how a novel Bayesian LCA, signature generation, and covariate reduction can be used as objective approaches to generate hypotheses for treatment. Given that the identification of prognostic and predictive biomarkers and clinical characteristics in colorectal cancers is an active area of research, this study shows how the development and application of statistical methods contributes to the retrospective analysis of trials. The ability to model and quantify the evidence for putative patient stratifications is therefore a crucial initial step towards identifying and validating strategies for targeting therapies.

## Funding

This work was supported by the CRUK UCL Centre (C416/A25145), CRUK City of London Centre (C7893/A26233), and CRUK KCL-UCL Comprehensive Cancer Imaging Centre (CRUK & EPSRC) in association with the MRC and DoH (C1519/A16463 and C1519/A10331); KCL Breast Cancer Now Unit (KCL 06/07); EU FP7 IMAGINT (EC: 259881); and Science Foundation Ireland (06/CE/B1129). We are grateful for the use of tissue samples and data from the COIN trial, funded by the MRC, Cancer Research UK (grant C1210/A4850) and Merck Serono, and coordinated by the MRC Clinical Trials Unit at UCL.

## Notes

The funders had no role in the study design, the collection, analysis and interpretation of the data, the writing of the manuscript, and the decision to publish.

PRB has received consultancy fees from Oxford Optronix Ltd TN wishes to declare research funding from AstraZeneca and Daiichi Sankyo. ACCC and MR are directors of Saddle Point Science Ltd. TM was in receipt of honoraria and travel expenses from Merck Serono. RA received travel expenses and honoraria from Merck Serono, Servier, and Amgen. DH has stock or other ownership interests in Novartis and Roche. He has also received research funding from Merck Serono and travel, accommodations, or expenses from Celgene.

GW, KL, JB, MR-J, DF, JD, FG, IDCT, LB, RK, and BV have no conflicts of interest to declare.

## Supplementary Material

djz231_supplementary_data
