# Supplementary material for: HER2-HER3 Heterodimer Quantification by FRET-FLIM and Patient Subclass Analysis of the COIN Colorectal Trial
Source: J Natl Cancer Inst. 2019 Dec 18;112(9):944–54. doi: 10.1093/jnci/djz231 (PMC7492762; doi:10.1093/jnci/djz231)
Supplement: djz231_supplementary_data [file djz231_supplementary_data.pdf]

HER2-HER3 heterodimer quantification by FRET-FLIM and patient subclass analysis of the COIN colorectal trial

Paul R Barber et al.

SUPPLEMENTAL MATERIAL

Supplementary Methods

Direct fluorophore labelling of antibodies

The anti-HER3 (clone B9A11, recognise intracellular epitope) antibody was purchased from Monogram Biosciences Inc. and the anti-HER2 (clone e2-4001+3B5, recognise intracellular epitope) antibody was purchased from ThermoScientific Ltd. They were directly labelled according to the manufacturer's protocols with Alexa546 (X546) and Cy5, respectively.

These primary antibodies were directly labelled with fluorophore at particular dye/protein (D/P) ratio for optimal performance of the FLIM-FRET assay (donor 1:1 and acceptor 1:3), as previously described[1]. The anti-HER3 IgG was labelled with Alexa546 (X546) (donor fluorophore) at D/P ratio ~1.0 and anti-HER2 was labelled with Cy5 at D/P ratio ~3-4 (making it suitable as an acceptor in the assay), while retaining antigen specificity in FFPE samples (Supplementary Fig.S1 for images of these labels).

FLIM Imaging and calculation of FRET Efficiency

Time-domain fluorescence lifetime images were acquired via time correlated single photon counting (TCSPC) at a resolution of 256 by 256 pixels, with 256 time bins and 100 frames accumulated over 300 seconds, via excitation and emission filters suitable for the detection of Alexa546 fluorescence (Excitation filter: Semrock FF01-540/15-25; Beam Splitter: Edmund 48NT-392 30R/70T; Emission filter: Semrock FF01-593/40-25). For technical convenience, those FLIM images were acquired through the emission channel of a UV filter cube (Long pass emission filter >420 nm). Conventional wide field fluorescence images were acquired with filter cubes for Alexa546 (Excitation 530-560 nm, emission 573-648 nm) and Cy5 (Excitation 590-650 nm, emission 663-738 nm), at a resolution of 1024 by 1024 pixels on a CCD camera (Hamamatsu 1394 ORCA-ERA), with an exposure time of typically 100-500 ms.

For each sample a 'donor' image and a 'donor with acceptor' image were acquired from serial sections, of the same area of the tissue core. FLIM analysis was performed with the TRI2 software (v2.7.8.9, Gray Institute, Oxford) as described previously[2-4]. Interfering effects of autofluorescence were minimised using processing with lifetime filtering algorithm[5].

The FRET efficiency (FRETeff) for each tissue sample region of interest was calculated according to the equation FRETeff=1–(τ_{DA}/τ_{D}), where τ_{D} is the average lifetime of Alexa546 in the absence of Cy5 from the donor image and τ_{DA} is the average lifetime of Alexa546 in the presence of Cy5, from the 'donor with acceptor' image.

FRET efficiency multiplied by HER3 fluorescence intensity, on a pixel-by-pixel basis, (FRETxHER3pbp) representing the amount of HER3 dimerized to HER2, was calculated by multiplying the image pixel FRET efficiency value by the image pixel fluorescence intensity of the Alexa546, from the 'donor with acceptor' image (corrected for fluorescence excitation intensity and imaging parameters). The average value from all the pixels was used to represent each sample.

Data Curation

Image metadata and image-derived values from multiple tissue microarrays (TMAs) were curated and inspected. Individual FLIM images were examined by eye and those of bad quality were rejected before values were calculated and the final set of patients were selected (approximately 2% of images were rejected). Patients samples were not randomised onto the TMAs resulting in some spatial patterning of FRET values on some of the TMA slides.

Multiple tissue samples from single patients were present on the 12 TMAs resulting in up to 4 measurements of FRET and FRET x HER3 per patient. In each case the maximum measured value was taken forward for further analysis. This was because the variability arises mostly from tissue and tumor heterogeneity and the maximum recorded signal indicates the most significant tissue area.

Bayesian latent class survival modelling

Bayesian latent class analysis was performed using ALPACA v0.2.15. The model and its implementation have been described previously[6]. Optimal models were reported on the basis of eight runs over possible model combinations M=1-3, L=1-4, K=1-10. Modelling progression-free survival using FRET imaging variables, treatment arm and KRAS mutation, there was evidence of two novel latent groups with *lnZ* (two groups) = -2418.1, *lnZ* (one group) = -2419.8 (Z ratio = 5.5), respectively. Within these groups the analysis determines the HR associated with each covariate (Table 2). The analysis also determines the residual risk (frailty) factor associated with class membership, from which the HR associated with one

class versus another can be determined. The risk factors for the 2 classes were -2.57 and -1.57, leading to the following calculation of this HR:

HR = exp(-2.57 - (-1.57)) = 0.4 (1 S.F.)

Modelling the subset of 189 patients in treatment arm A, who did not receive cetuximab, also revealed 2 latent groups, but there was insufficient statistical power to resolve significant covariate HRs in this case. *lnZ* (two groups) = -1177.4, *lnZ* (one group) = -1178.5 (Z ratio = 3.0).

Statistical Analysis

All survival analysis was performed in R using the survival package. Code is available online:

https://github.com/paulbarber/COINPaperFigures

Median OS in FRET cohort patients by OS-based latent class

Output from R survival package.

```
Call: survfit(formula = SurvObj.os ~ OS.Class + TRT, data = data)

                      n events median 0.95LCL 0.95UCL
OS.Class=1, TRT=A    22     15   1447    1230      NA
OS.Class=1, TRT=B    40     24   1668    1531      NA
OS.Class=2, TRT=A   167    166    474     430     515
OS.Class=2, TRT=B   169    168    455     393     521
```

Median OS in all FRET cohort patients

Output from R survival package.

```
Call: survfit(formula = SurvObj.os ~ TRT, data = data)

        n events median 0.95LCL 0.95UCL
TRT=A 189    181    505     471     597
TRT=B 209    192    581     492     678
```

Class membership comparison permutations test

Calculate the means and standard deviations from 100,000 random group assignments with the same number per subclass as the real data. Also do permutations test and see how many random group are as, or more, extreme as the real data.

Random group statistics:

```
[1] "Class number mean and sd."
     [,1]         [,2]         [,3]
[1,] "4.2±1.82"  "13.4±2.99" "44.4±3.26"
[2,] "22.8±1.82" "72.6±2.99" "241±3.26"
[1] "p-values."

              1            2            3
  1 2.706736e-30 4.753353e-10 1.467638e-33
  2 2.706736e-30 4.753353e-10 1.467638e-33
```

Compare Class 1-Class 1 overlap: random assignment results in 4.2 ± 1.82 patients in Class 1 of both assignments, real data had 25 patients in Class 1 of both analyses.

Of 100,000 permutations, none were as extreme as the real data.

The 115 baseline covariates, data types and statistics

Covariate name	statistics	Description
1: TRT:A	type=int missing= 0 av=0.500000 sd=0.500000 range=[0,1]	Treatment Arm
2: CHEMO:OxMdG	type=int missing= 0 av=0.343558 sd=0.474896 range=[0,1]	Chemotherapy regimen
3: AGE	type=int missing= 0 av=62.342945 sd=9.788603 range=[22,87]	Age (at randomisation)
4: SEX:Female	type=int missing= 0 av=0.344172 sd=0.475097 range=[0,1]	Sex of patient
5: HT	type=int missing= 8 av=170.099260 sd=9.325562 range=[131,198]	Height
6: HT Missing	type=int missing= 0 av=0.004908 sd=0.069885 range=[0,1]	
7: WT	type=float missing= 7 av=76.051571 sd=15.979053 range=[40.0,158.0]	Weight
8: WT Missing	type=int missing= 0 av=0.004294 sd=0.065391 range=[0,1]	
9: WHO	type=int missing= 0 av=0.615951 sd=0.623460 range=[0,2]	WHO performance status (baseline)
10: PRT:No	type=int missing= 6 av=0.969212 sd=0.172743 range=[0,1]	Prior radiotherapy
11: PRT Missing	type=int missing= 0 av=0.003681 sd=0.060559 range=[0,1]	
12: PSURG:No	type=int missing= 6 av=0.832512 sd=0.373411 range=[0,1]	Prior surgery
13: PSURG Missing	type=int missing= 0 av=0.003681 sd=0.060559 range=[0,1]	
14: ADJCH:>1m and <6m ago	type=int missing= 0 av=0.041718 sd=0.199944 range=[0,1]	Adjuvant chemotherapy
15: ADJCH:>6 months ago	type=int missing= 0 av=0.160123 sd=0.366720 range=[0,1]	
16: ADJCH:No	type=int missing= 0 av=0.747239 sd=0.434595 range=[0,1]	
17: ADJCH:Yes (unspecified)	type=int missing= 0 av=0.050920 sd=0.219835 range=[0,1]	
18: TSTAT:Local recurrence	type=int missing= 0 av=0.053988 sd=0.225993 range=[0,1]	Baseline tumour status
19: TSTAT:Resected	type=int missing= 0 av=0.530675 sd=0.499058 range=[0,1]	
20: TSTAT:Unresected/unresectable	type=int missing= 0 av=0.415337 sd=0.492780 range=[0,1]	
21: SUMLES	type=int missing= 24 av=106.648817 sd=85.160269 range=[1,625]	Sum of longest diameter
22: SUMLES Missing	type=int missing= 0 av=0.014724 sd=0.120446 range=[0,1]	
23: NOLES	type=int missing= 23 av=2.935905 sd=1.954109 range=[1,30]	No of lesions
24: NOLES Missing	type=int missing= 0 av=0.014110 sd=0.117946 range=[0,1]	
25: left:Left-sided	type=int missing= 32 av=0.712140 sd=0.452765 range=[0,1]	Sidedness of primary tumour
26: left Missing	type=int missing= 0 av=0.019632 sd=0.138732 range=[0,1]	
27: MLIV:No	type=int missing= 0 av=0.250307 sd=0.433190 range=[0,1]	Liver metastases
28: MLNG:No	type=int missing= 0 av=0.593252 sd=0.491227 range=[0,1]	Lung metastases
29: MNODE:No	type=int missing= 0 av=0.558282 sd=0.496592 range=[0,1]	Nodal metastases status
30: MOTH:No	type=int missing= 0 av=0.834356 sd=0.371761 range=[0,1]	Other metastases
31: MPERI:No	type=int missing= 0 av=0.852147 sd=0.354954 range=[0,1]	Peritoneal metastases
32: metsites t	ype=int missing= 0 av=1.911656 sd=0.881743 range=[0,5]	Number of metastatic sites
33: mlivonly:No	type=int missing= 0 av=0.774233 sd=0.418086 range=[0,1]	Liver-only metastases
34: metscat:Metachronous	type=int missing= 18 av=0.303350 sd=0.459705 range=[0,1]	Timing of metastases
35: metscat Missing	type=int missing= 0 av=0.011043 sd=0.104504 range=[0,1]	
36: diagtime	type=int missing= 18 av=82.374690 sd=188.582800 range=[-344,2359]	Days since metastatic diagnosis at randomisation
37: diagtime Missing	type=int missing= 0 av=0.011043 sd=0.104504 range=[0,1]	
38: nEREG	type=float missing=678 av=-3.161197 sd=2.259342 range=[-13.42,1.35]	EREG Cq value (negated)
39: nEREG Missing	type=int missing= 0 av=0.415951 sd=0.492885 range=[0,1]	
40: nAREG	type=float missing=678 av=-2.822742 sd=1.595901 range=[-12.84,1.45]	AREG Cq value (negated)
41: nAREG Missing	type=int missing= 0 av=0.415951 sd=0.492885 range=[0,1]	
42: RAS:Mutation	type=int missing=322 av=0.466361 sd=0.498867 range=[0,1]	KRAS mutation status
43: RAS Missing	type=int missing= 0 av=0.197546 sd=0.398148 range=[0,1]	

44: BRAF:Mutation type=int missing=336 av=0.078825 sd=0.269466 range=[0,1]	BRAF mutation status
45: BRAF Missing type=int missing= 0 av=0.206135 sd=0.404529 range=[0,1]	
46: MSI:MSI type=int missing=608 av=0.044031 sd=0.205165 range=[0,1]	Microsatellite stability status
47: MSI Missing type=int missing= 0 av=0.373006 sd=0.483604 range=[0,1]	
48: PIK3CA:Mutation type=int missing=367 av=0.123515 sd=0.329028 range=[0,1]	PIK3CA mutation status
49: PIK3CA Missing type=int missing= 0 av=0.225153 sd=0.417683 range=[0,1]	
50: WBC type=float missing= 6 av=8.982204 sd=3.992375 range=[2.4,90.0]	White blood cell count
51: WBC Missing type=int missing= 0 av=0.003681 sd=0.060559 range=[0,1]	
52: NEUT type=float missing= 6 av=6.294766 sd=3.576336 range=[1.6,66.3]	Neutrophil count
53: NEUT Missing type=int missing= 0 av=0.003681 sd=0.060559 range=[0,1]	
54: PLT type=int missing= 9 av=356.308452 sd=132.582668 range=[116,999]	Platelet count
55: PLT Missing type=int missing= 0 av=0.005521 sd=0.074101 range=[0,1]	
56: BIL type=int missing= 6 av=9.454433 sd=4.810103 range=[1,41]	Bilirubin
57: BIL Missing type=int missing= 0 av=0.003681 sd=0.060559 range=[0,1]	
58: ALKP type=int missing= 6 av=191.666872 sd=176.731603 range=[27,1497]	Alkaline phosphatase
59: ALKP Missing type=int missing= 0 av=0.003681 sd=0.060559 range=[0,1]	
60: HEPFNC:ALT type=int missing= 6 av=0.649015 sd=0.477278 range=[0,1]	Hepatobiliary function test
61: HEPFNC Missing type=int missing= 0 av=0.003681 sd=0.060559 range=[0,1]	
1262: hepato type=int missing= 6 av=30.694581 sd=20.686621 range=[2,234]	Hepatobiliary function (U/l)
63: hepato Missing type=int missing= 0 av=0.003681 sd=0.060559 range=[0,1]	
64: M RENAL:Creatinine clearance type=int missing= 10 av=0.882716 sd=0.321758 range=[0,1]	Which renal function
65: M RENAL Missing type=int missing= 0 av=0.006135 sd=0.078085 range=[0,1]	
66: renal type=float missing= 11 av=86.728351 sd=32.026323 range=[32.0,760.0]	Renal function (ml/min)
67: renal Missing type=int missing= 0 av=0.006748 sd=0.081871 range=[0,1]	
68: SNAUS0 type=int missing= 14 av=0.112005 sd=0.367901 range=[0,3]	Nausea at baseline (CTC grade)
69: SNAUS0 Missing type=int missing= 0 av=0.008589 sd=0.092278 range=[0,1]	
70: SVOM0 type=int missing= 12 av=0.039555 sd=0.237764 range=[0,3]	Vomiting at baseline (CTC grade)
71: SVOM0 Missing type=int missing= 0 av=0.007362 sd=0.085485 range=[0,1]	
72: SANORX0 type=int missing= 19 av=0.232154 sd=0.538508 range=[0,3]	Anorexia at baseline (CTC grade)
73: SANORX0 Missing type=int missing= 0 av=0.011656 sd=0.107334 range=[0,1]	
74: SALOPC0 type=int missing= 14 av=0.009901 sd=0.105074 range=[0,2]	Alopecia at baseline (CTC grade)
75: SALOPC0 Missing type=int missing= 0 av=0.008589 sd=0.092278 range=[0,1]	
76: SPAIN0 type=int missing= 15 av=0.547988 sd=0.739019 range=[0,4]	Pain at baseline (CTC grade)
77: SPAIN0 Missing type=int missing= 0 av=0.009202 sd=0.095487 range=[0,1]	
78: SSTOM0 type=int missing= 14 av=0.021040 sd=0.147765 range=[0,2]	Stomatitis at baseline (CTC grade)
79: SSTOM0 Missing type=int missing= 0 av=0.008589 sd=0.092278 range=[0,1]	
80: SNAILC0 type=int missing= 22 av=0.008706 sd=0.092901 range=[0,1]	Nail changes at baseline (CTC grade)
81: SNAILC0 Missing type=int missing= 0 av=0.013497 sd=0.115390 range=[0,1]	
82: SDIAR0 type=int missing= 18 av=0.137097 sd=0.414308 range=[0,3]	Diarrhoea at baseline (CTC grade)
83: SDIAR0 Missing type=int missing= 0 av=0.011043 sd=0.104504 range=[0,1]	
84: SLETH0 type=int missing= 15 av=0.489164 sd=0.653431 range=[0,3]	Lethargy at baseline (CTC grade)
85: SLETH0 Missing type=int missing= 0 av=0.009202 sd=0.095487 range=[0,1]	
86: SPLT0 type=int missing= 11 av=0.011118 sd=0.110588 range=[0,2]	Platelets at baseline (CTC grade)
87: SPLT0 Missing type=int missing= 0 av=0.006748 sd=0.081871 range=[0,1]	
88: SHB0 type=int missing= 9 av=0.254164 sd=0.560517 range=[0,4]	Haemaglobin at baseline (CTC grade)
89: SHB0 Missing type=int missing= 0 av=0.005521 sd=0.074101 range=[0,1]	
90: SWBC0 type=int missing= 9 av=0.006169 sd=0.078301 range=[0,1]	WBC at baseline (CTC grade)
91: SWBC0 Missing type=int missing= 0 av=0.005521 sd=0.074101 range=[0,1]	
92: SNEUT0 type=int missing= 9 av=0.005552 sd=0.074305 range=[0,1]	Neutrophils at baseline (CTC grade)
93: SNEUT0 Missing type=int missing= 0 av=0.005521 sd=0.074101 range=[0,1]	
94: SRASH0 type=int missing= 14 av=0.019183 sd=0.150093 range=[0,2]	Skin rash at baseline (CTC grade)
95: SRASH0 Missing type=int missing= 0 av=0.008589 sd=0.092278 range=[0,1]	
96: SHFS0 type=int missing= 18 av=0.003102 sd=0.055607 range=[0,1]	Hand-foot syndrome at baseline (CTC grade)
97: SHFS0 Missing type=int missing= 0 av=0.011043 sd=0.104504 range=[0,1]	
98: SVPAIN0 type=int missing= 30 av=0.001875 sd=0.043261 range=[0,1]	Vein pain at baseline (CTC grade)
99: SVPAIN0 Missing type=int missing= 0 av=0.018405 sd=0.134410 range=[0,1]	
100: SPNP0 type=int missing= 15 av=0.030960 sd=0.176747 range=[0,2]	Peripheral neuropathy at baseline (CTC grade)
101: SPNP0 Missing type=int missing= 0 av=0.009202 sd=0.095487 range=[0,1]	
102: SHYPMG0 type=int missing=447 av=0.018597 sd=0.135096 range=[0,1]	Hypomagnesaemia at baseline (CTC grade)
103: SHYPMG0 Missing type=int missing= 0 av=0.274233 sd=0.446127 range=[0,1]	
104: site type=int missing= 0 av=54.741104 sd=68.245437 range=[1,510]	Clinical site/centre identifier
105: nsite type=int missing= 0 av=24.995092 sd=14.482695 range=[1,56]	Total no. of randomisations at this clincal site
106: RMARK:No type=int missing= 70 av=0.157051 sd=0.363849 range=[0,1]	Was a raised tumour marker test performed?
107: RMARK Missing type=int missing= 0 av=0.042945 sd=0.202733 range=[0,1]	
108: CEA type=int missing=372 av=686.988871 sd=2847.921778 range=[0,55000]	Value of CEA tumour marker
109: CEA Missing type=int missing= 0 av=0.228221 sd=0.419686 range=[0,1]	
110: CA19 9 type=int missing=1543 av=2946.000000 sd=12977.193552 range=[10,97513]	Value of CA19 tumour marker
111: CA19 9 Missing type=int missing= 0 av=0.946626 sd=0.224779 range=[0,1]	
112: CA125 type=int missing=1628 av=170.500000 sd=48.500000 range=[122,219]	Value of CA125 tumour marker
113: CA125 Missing type=int missing= 0 av=0.998773 sd=0.035007 range=[0,1]	
114: CRP type=int missing=1626 av=66.750000 sd=29.911327 range=[36,114]	Value of CRP tumour marker
115: CRP Missing type=int missing= 0 av=0.997546 sd=0.049477 range=[0,1]	

Supplementary Figures

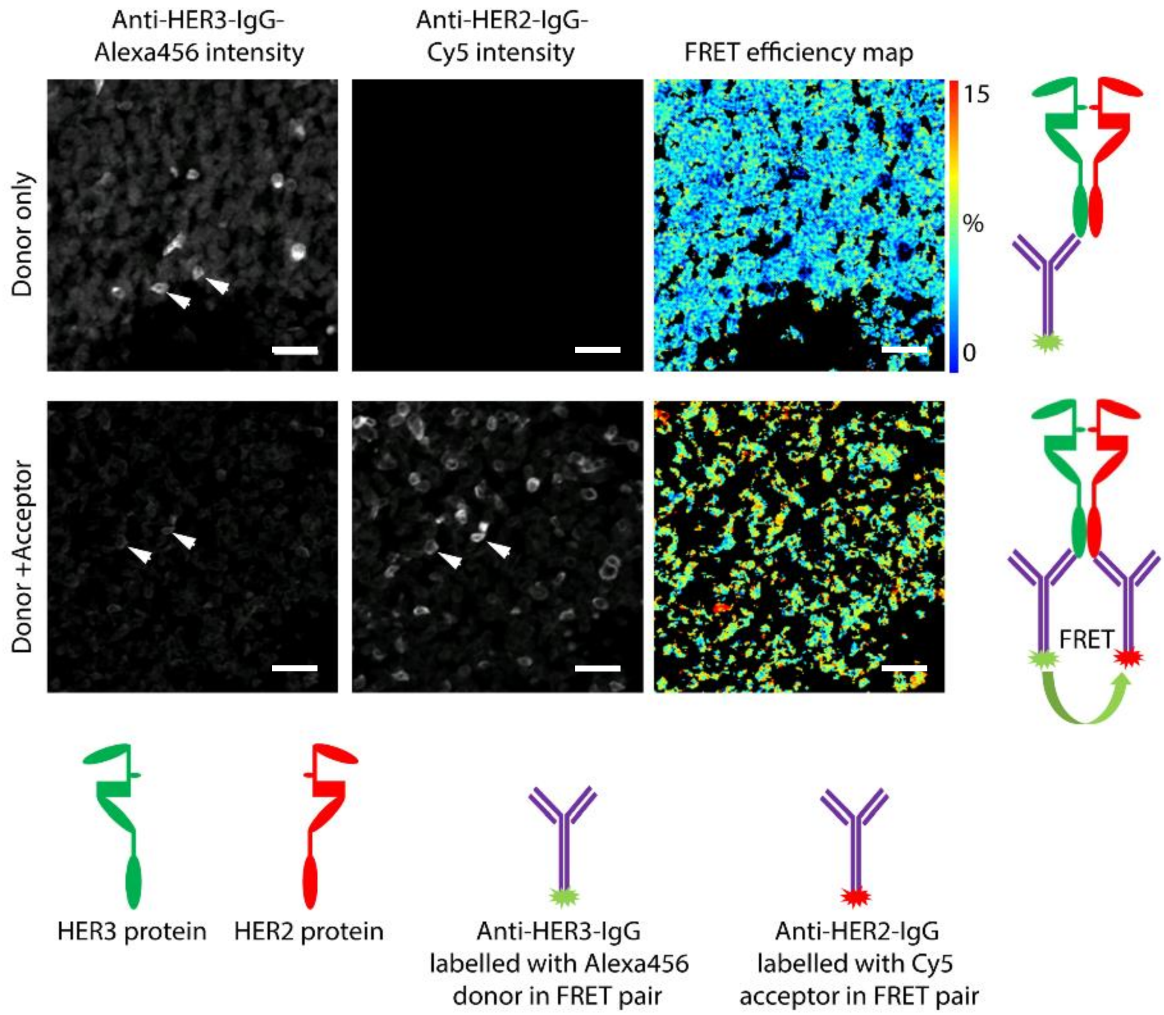

Supplementary Figure 1. Validation of HER2-HER3 dimer FRET-FLIM assay in FFPE cell pellets

SKBR3 cells were transfected with HER2 and HER3 encoding DNA at ratio 3:1, fixed in buffered formaline saline and put into paraffin block to mimic patients sample fixation procedure. After antigen retrieval, cells were stained with anti-HER3-Alexa546 (donor) and anti-HER2-Cy5 (acceptor) antibodies. Images were acquired as described in the main text. Scale bar = 50μm.

Supplementary Figure 2. Distribution of FRET efficiency for patients assigned to the two OS latent groups. (box contains LQ to UQ). The FRET efficiency distributions of the patients retrospectively assigned to the two novel latent groups reveals that Class 2 has a statistically significantly higher degree of dimerization on average (unpaired t-test with Welch's correction). All tests were two-sided.

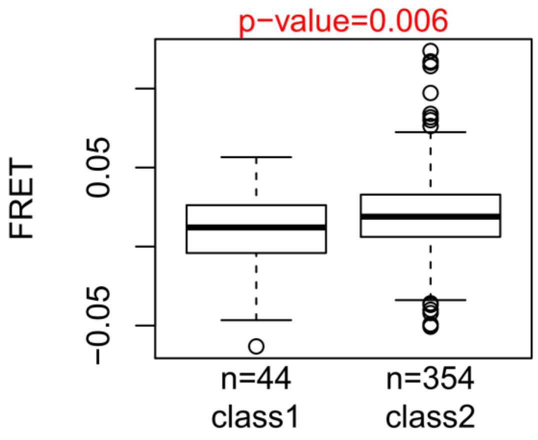

See: https://github.com/paulbarber/COINPaperFigures

Supplementary Figure 3. Latent Class Analysis on the 1,232 patients from the full cohort that are non-overlapping with the FRET cohort. Similar classes are found as with the full 1,630 patients with differing treatment responses (see HR for TRT.B: 0.42 in Class 1, 1.2 in Class 2 and 1.5 in Class 3).

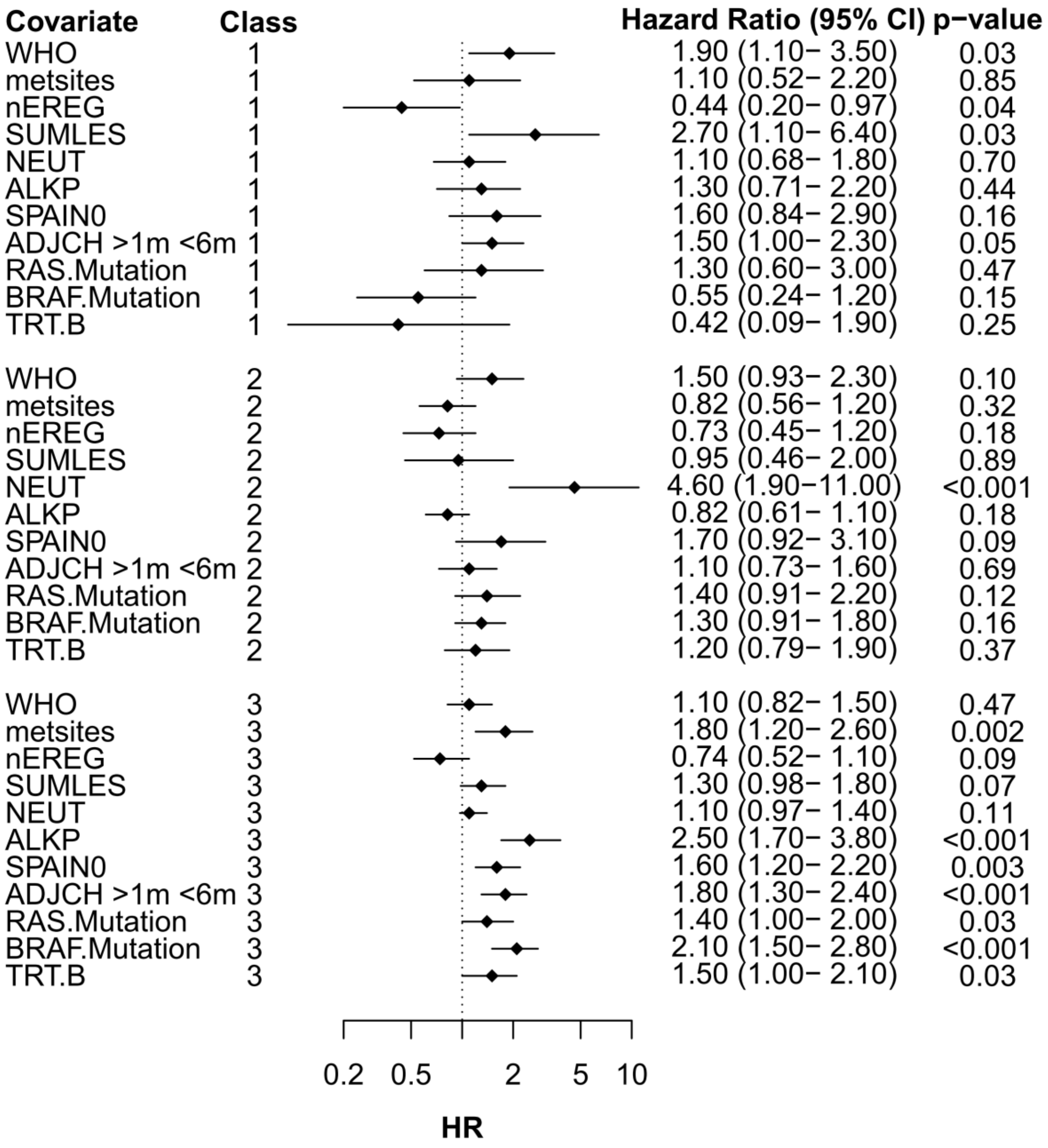

Supplementary Figure 4. *PIK3CA* mutation an indicator of positive response in the FRET cohort

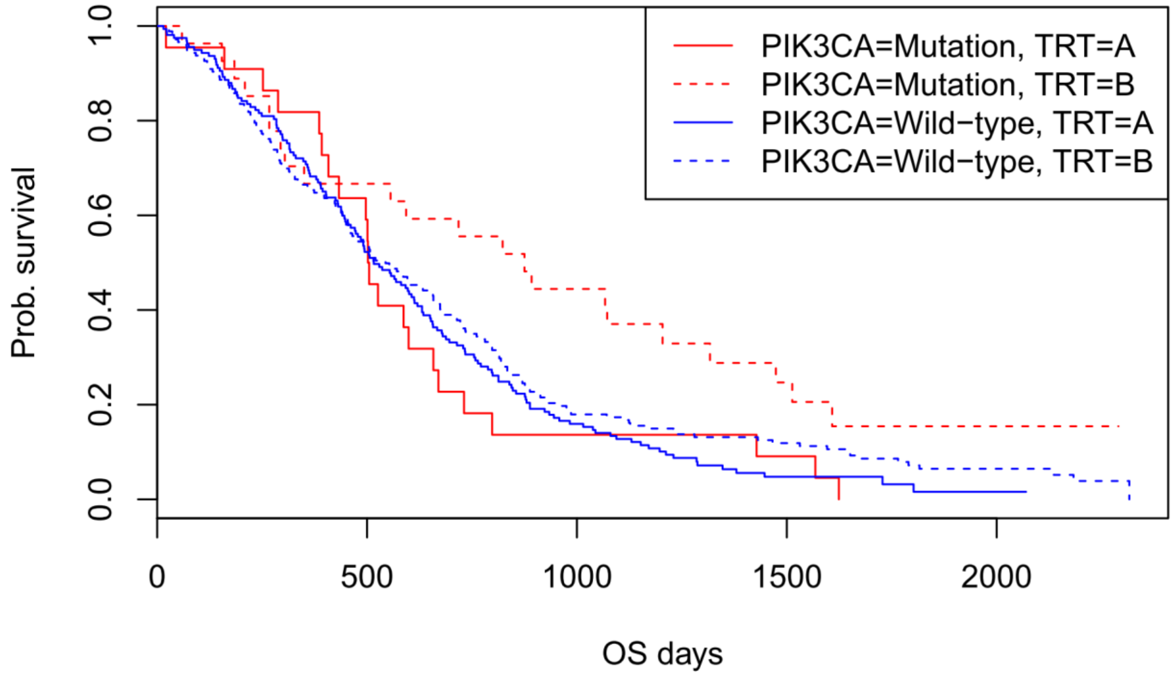

Output from R survival package.

```
Call: survfit(formula = SurvObj.os ~ PIK3CA + TRT, data = data)

   15 observations deleted due to missingness
                           n events median 0.95LCL 0.95UCL
PIK3CA=Mutation, TRT=A    22     22    504     433     670
PIK3CA=Mutation, TRT=B    27     22    875     556    1474
PIK3CA=Wild-type, TRT=A  158    150    516     471     613
PIK3CA=Wild-type, TRT=B  176    164    544     458     658
```

```
Call:
survdiff(formula = SurvObj.os[PIK3CA == "Mutation"] ~ TRT[PIK3CA ==
    "Mutation"], data = data)

n=49, 15 observations deleted due to missingness.

                             N Observed Expected (O-E)^2/E (O-E)^2/V
TRT[PIK3CA == "Mutation"]=A 22       22     15.4      2.78      4.52
TRT[PIK3CA == "Mutation"]=B 27       22     28.6      1.51      4.52

 Chisq= 4.5  on 1 degrees of freedom, p= 0.03
```

See: https://github.com/paulbarber/COINPaperFigures

Supplementary Figure 5. The interplay of FRET alone and within the Class Prediction Signature (c.f. Figure 5A). High FRET value is good for some patients but not for others. **A**) Plot of Signature Residual Score (equal to the class prediction signature score minus the FRET component) against FRET Efficiency value for all 550 FRET cohort patients. Patient groups were formed using the optimal threshold for class prediction (blue line) and the median FRET value (dot colours). Inset are the survival curves split by treatment for each group, with the median survival indicated as a number of days. For patients with a low Signature Residual Score, a higher FRET means moving towards a treatment effect in Group iv (log rank p-value=0.02) (Higher FRET moves from Group iii to Group iv; c.f. Figure 2 and Figure 3 for LCA Class 1). All other covariates in the signature were investigated in this way and FRET is the only one that indicates a treatment effect. For patients with an average residual score, a higher FRET will tend to push towards Class 2 where prognosis is worse (Group iii to Group i or ii; c.f. Table 2 and Figure 5). For patients with a high residual score, FRET has a correlation with outcome as predicted in LCA Class 2 (Group i to Group ii; c.f. Figure 3). **B**) Survival curves for the 4 groups and table of patient/event numbers and median survival.

A

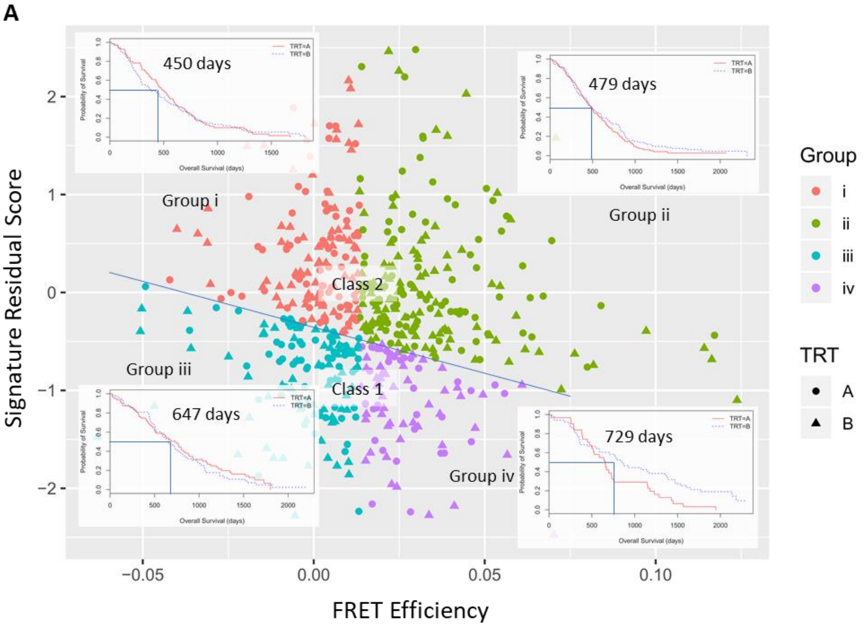

B

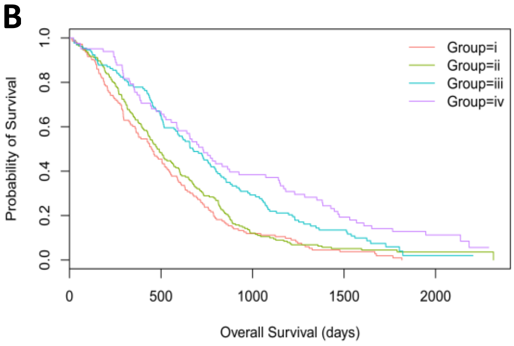

	n	events	median	0.95LCL	0.95UCL
Group=i	143	141	450	364	536
Group=ii	193	184	479	430	571
Group=iii	131	120	674	590	798
Group=iv	82	73	729	587	1072

REFERENCES

1. Parsons M, Ng T: Intracellular coupling of adhesion receptors: molecular proximity measurements. Methods Cell Biol 69:261-78, 2002
2. Rowley MI, Barber PR, Coolen ACC, et al: Bayesian analysis of fluorescence lifetime imaging data. Multiphoton Microscopy in the Biomedical Sciences Xi 7903, 2011
3. Rowley MI, Coolen ACC, Vojnovic B, et al: Robust Bayesian Fluorescence Lifetime Estimation, Decay Model Selection and Instrument Response Determination for Low-Intensity FLIM Imaging. PLOS ONE 11:e0158404, 2016
4. Barber PR, Ameer-Beg SM, Gilbey J, et al: Global and pixel kinetic data analysis for FRET detection by multi-photon time-domain FLIM. Proc. SPIE 5700:171-181 2005
5. Barber PR, Tullis IDC, Rowley MI, et al: The Gray Institute open microscopes applied to radiobiology and protein interaction studies. Proc. SPIE 8949:89490D–17, 2014
6. Rowley M, Garmo H, Van Hemelrijck M, et al: A latent class model for competing risks. Statistics in Medicine:10.1002/sim.7246, 2017
